# One-step hydrothermal synthesis of CeVO_4_/bentonite nanocomposite as a dual-functional photocatalytic adsorbent for the removal of methylene blue from aqueous solutions

**DOI:** 10.1038/s41598-024-65793-9

**Published:** 2024-06-27

**Authors:** Hajar Farhadi, Mehdi Mousavi-Kamazani, Narjes Keramati, Sanaz Alamdari

**Affiliations:** Department of Nanotechnology, Faculty of New Sciences and Technologies, Semnan University, Semnan, Iran

**Keywords:** Photocatalytic adsorbent, CeVO_4_/bentonite, Nanocomposite, Hydrothermal, Methylene blue, Chemistry, Nanoscience and technology, Physics

## Abstract

Cerium vanadate/modified bentonite (CeVO_4_/mbt) nanocomposite with different composition percentages was synthesized through a simple one-step hydrothermal method at 180 ℃, and then its photocatalytic activity was evaluated by decolorizing methylene blue (MB) in an aqueous solution under light exposure. In order to increase the surface area as an important parameter in photocatalytic processes, bentonite was modified by ball mill method. The structural and optical properties of the synthesized composites were determined by XRD, FT-IR, DRS, FESEM, EDS, and BET measurements. XRD and EDS results confirmed the successful synthesis of pure CeVO_4_. FESEM images and EDS mapping showed a proper distribution of rice-like CeVO_4_ nanoparticles on bentonite. The removal efficiency of MB with only 0.1 g of CeVO_4_/mbt nanocomposite in 15 min was about 99%, which is significant compared to neat bentonite and pure CeVO_4_ with efficiency of 30% and 57%. The mentioned nanocomposite followed the first-order kinetics, had a reaction rate constant equal to 0.1483 min^–1^, and showed acceptable stability in five consecutive cycles.

## Introduction

Water pollution by industries such as textile, paint, petrochemical is one of the biggest problems in the world and has irreparable consequences for mankind^1,2^. Pollution of water resources is important on a global scale and has irreparable consequences for mankind^3,4^. One of the most dangerous water pollutants are all kinds of chemicals, which enter the nature and perform numerous reactions with other substances, and will eventually have an adverse effect^5,6^. Dyes are one of the important chemical pollutants in wastewater^1^. Meanwhile, methylene blue (MB) is one of the cationic dyes that was widely used in textiles, leather, paper, printing, cosmetics, paints, plastics, food, and medicine^7^. Adverse effects of exposure to this dye include irritation of the skin, throat and stomach, esophagus, nausea, digestive pains, diarrhea, vomiting, and high blood pressure^8^. In recent years, different processes have been used to remove various dyes, including adsorption, photocatalytic, ion exchange, sedimentation, reverse osmosis, membrane filtration, and oxidation. Each of these methods has its own strengths and weaknesses^9–11^. As one of the advanced environmental purification technologies, the photocatalytic degradation has attracted more attention in creating an option for the problems of dyes pollution. Existence of an ideal photocatalyst that can effectively absorb light and use the photo-generated charge carriers for the reduction reaction is still a challenge^12–15^. This process involves the absorption of photons that have an energy (hν) greater than the photocatalyst's bandgap, and as a result, electrons (e^–^) are excited from its valence band to the conduction band, thus leading to the creation of a hole (h^+^). These excited e^–^ and h^+^ then react with available oxygen and water to form the powerful hydroxyl radical, the key oxidizing species^15^. A unique approach to remove dyes by photocatalytic degradation process is the combination of two or more semiconductors such as g-C_3_N_4_, ZnO, Fe_2_O_3_, ZnIn_2_S_4_, ZnS, MoO_3_ with different energy levels^16,17^. Photocatalyst adsorbent is a combination of a photocatalyst such as TiO_2_, ZnO, WO_3_, Fe_2_O_3_ or CeVO_4_ and an adsorbent such as zeolite, activated carbon or activated alumina^18^. CeVO_4_ is a reductive photocatalyst with a bandgap of about 2.40 eV. Due to its accessibility, chemical stability, non-toxicity, and high photocatalytic activity, it has attracted great attention^19–21^. On the other hand, clay minerals such as bentonite (bt) are among the most widely used adsorbents for the removal of cationic pollutants^22^. Photocatalyst adsorbent materials are synthesized simultaneously and have the ability to adsorb and degrade dye molecules^18^. Phuruangrat et al. synthesized Dy-doped CeVO_4_ nanorods and investigated MB and RhB solutions. Degradation efficiency of 94 and 93% was achieved in 180 min, respectively^23^. Ren et al. synthesized photocatalyst (CeVO_4_/g-C_3_N_4_) via a facile mixing method. The photocatalytic performance of the CeVO_4_/g-C_3_N_4_ samples was evaluated by degrading MB under visible light irradiation. After 120 min of visible light irradiation, the degradation efficiency of MB using CeVO_4_ and CeVO_4_/g-C_3_N_4_ was 14.3 and 93.8%, respectively^24^. Bansal et al. investigated the nano-adsorbent photocatalytic performance of vanadium pentoxide with functionalized chitosan and the photocatalytic performance of the sample was determined under visible light irradiation and by degradation of MB dye. The degradation efficiency of MB was 94.8%^25^.

In this work, for the first time, cerium vanadate/bentonite nanocomposite with different percentages of compounds was synthesized by hydrothermal method and characterized by XRD, FT-IR, BET, EDS, FESEM, and DRS analyses. Then, the synthesized products were investigated as photocatalysts for methylene blue (MB) removal under visible light irradiation. Also, in order to improve the dye removal efficiency, another synthesis was carried out in which milled bentonite was used. Our next innovation is about the method of performing the photocatalytic process in such a way that the lamp is lit from the beginning. In common photocatalytic studies, the dye solution with the photocatalyst is placed in the dark until adsorption and desorption are balanced, then the lamp is turned on, but this method is not suitable for adsorbent-photocatalysts that exhibit high adsorption in the dark.

## Experimental

### Materials and instruments

All materials and chemical compounds used in this study including cerium nitrate hexahydrate (Ce(NO_3_)_3_.6H_2_O), ammonium metavanadate (NH_4_VO_3_), hydrazinium hydroxide (N_2_H_2_OH), nitric acid (HNO_3_(, sodium hydroxide (NaOH) were purchased from Merck and all chemicals were pure. Bentonite as a natural mineral was obtained from Negin Powder Semnan Company (Iran). Distilled water was used during the experiment.

The crystal structure of the material was determined by XRD (X-ray diffraction) (D8-Advance, Bruker, Germany) using Cu Kα radiation (λ = 1.5406 Å) in the 2θ range from 10 to 70°. Fourier transform infrared (FTIR, Magna-IR, spectrophotometer 550 Nicolet in KBr pellets ranging from 400 to 4000 cm^−1^) spectroscopy has been used to study the structure and chemical bonds of the molecule. FESEM (field-emission scanning electron microscope) images was recorded by MIRA3 FE-SEM. Nitrogen adsorption/desorption was done using Belsorp mini x device. BJH analysis was used to record the pore size distribution. The bandgap of the samples was determined using UV–visible diffuse reflection spectra (DRS) equipment (Shimadzu/UV3600Iplus).

### Synthesis of CeVO_4_/bentonite

**Figure 1:**
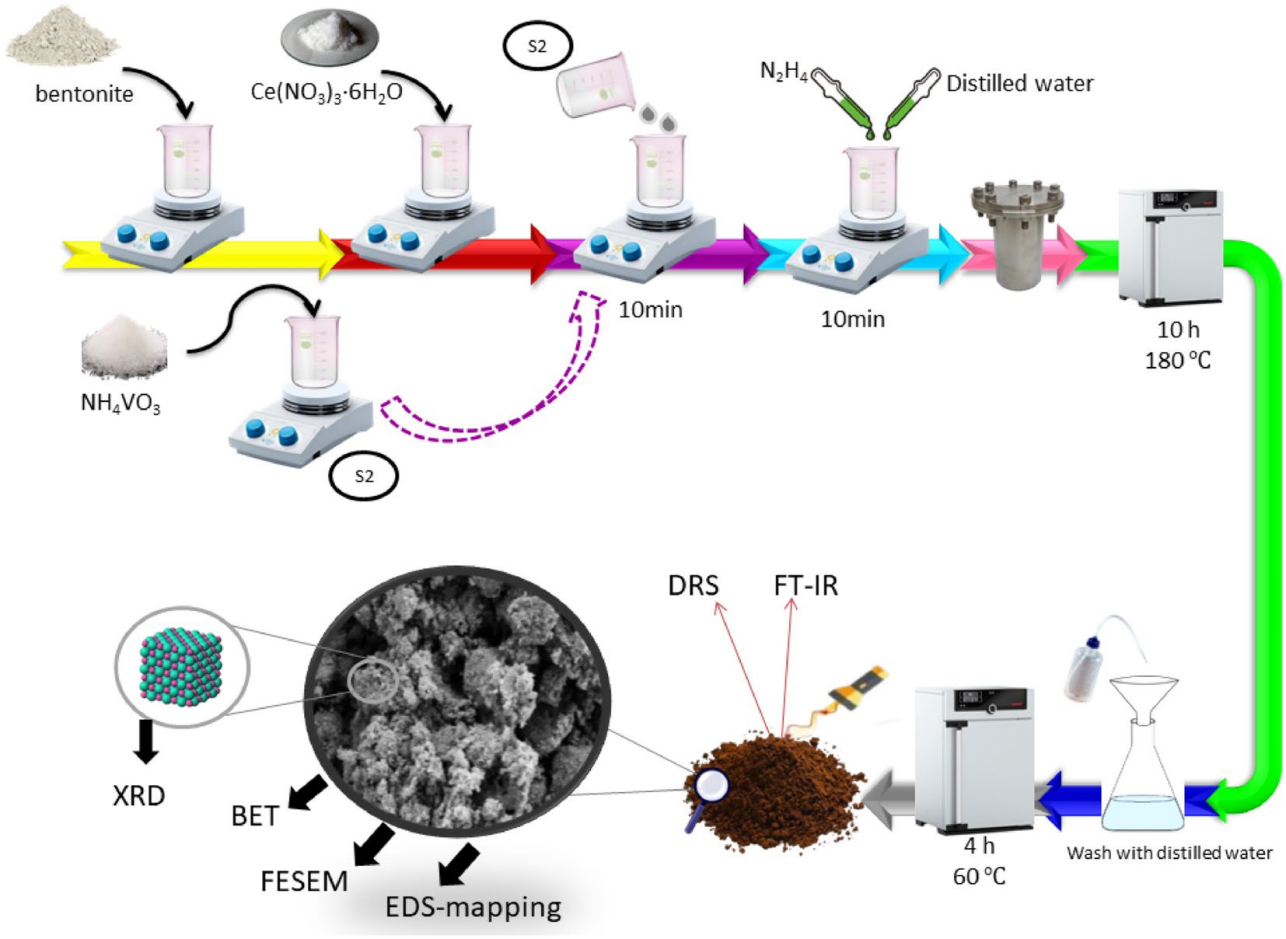
Synthesis steps of CeVO_4_/bentonite nanocomposite.

First, a certain amount of bentonite was dissolved in 30 ml of distilled water, then one mmol (0.43 g) of Ce(NO_3_)_3_.6H_2_O was added to it (first solution). Also, one mmol (0.116 g) of ammonium metavanadate (NH_4_VO_3_) was dissolved in 20 ml of distilled water (second solution). The second solution was added to the first solution and the resulting suspension was stirred on a stirrer for 10 min. Then, 2 ml of hydrazine and 8 ml of distilled water were added to the above solution until its pH reached about 8.5. After mixing on a stirrer for 10 min, the prepared suspension was transferred to an autoclave and kept at 180 ℃ for 10 h. The temperature and time were chosen based on the results of our previous work on the synthesis of pure CeVO_4_ nanostructures by hydrothermal method^26^. Finally, the autoclave was cooled to ambient temperature and the resulting precipitate was dried after several washings with distilled water at 60 °C for 4 h. The synthesized samples were named as CeVO_4_/bt X. The value of X was the weight of bt (0.25 and 0.5 g) which used in the synthesis. Bentonite was modified by ball mill method and used in CeVO_4_/bt composites preparation similar to the above procedure and the synthesis sample was named as CeVO_4_/mbt. The amount of bentonite used in the CeVO_4_/mbt sample was 0.5 g. To perform grinding, bentonite with a mass ratio of 1:10 (bentonite powder to steel ball) was poured into the 250 ml cup of the machine (35 g of bentonite, 350 g of metal balls). The cup was closed and placed inside the machine, and the machine was set to 500 rpm rotation speed and 6 h (See Fig. 1).

### Photocatalytic experiments

Photocatalytic experiments were performed by adding 0.1 g of photocatalyst to a specified volume of methylene blue solution in the reactor at room temperature, and HNO_3_ and NaOH solutions were used to adjust the pH. Finally, sampling was done and the photocatalyst was separated from the solution using a centrifuge, and the concentration of the samples was determined by UV–Vis spectrophotometer at 664 nm. Then the results were compared. The photocatalytic degradation efficiency of methylene blue was calculated using Eq. (1):

1 $$\text{Decolorization efficiency }\left(\text{\%}\right)=\frac{{C}_{0}-C}{{C}_{0}}\times 100.$$

C_0_ is the concentration of methylene blue at the initial moment on the surface of the photocatalyst and C is the concentration after visible light irradiation. In order to investigate the kinetics of the degradation reaction, samples were taken from the system at time intervals of 0, 5, 10 and 15 min and the concentration of methylene blue was determined.

## Results and discussion

### XRD studies

**Figure 2:**
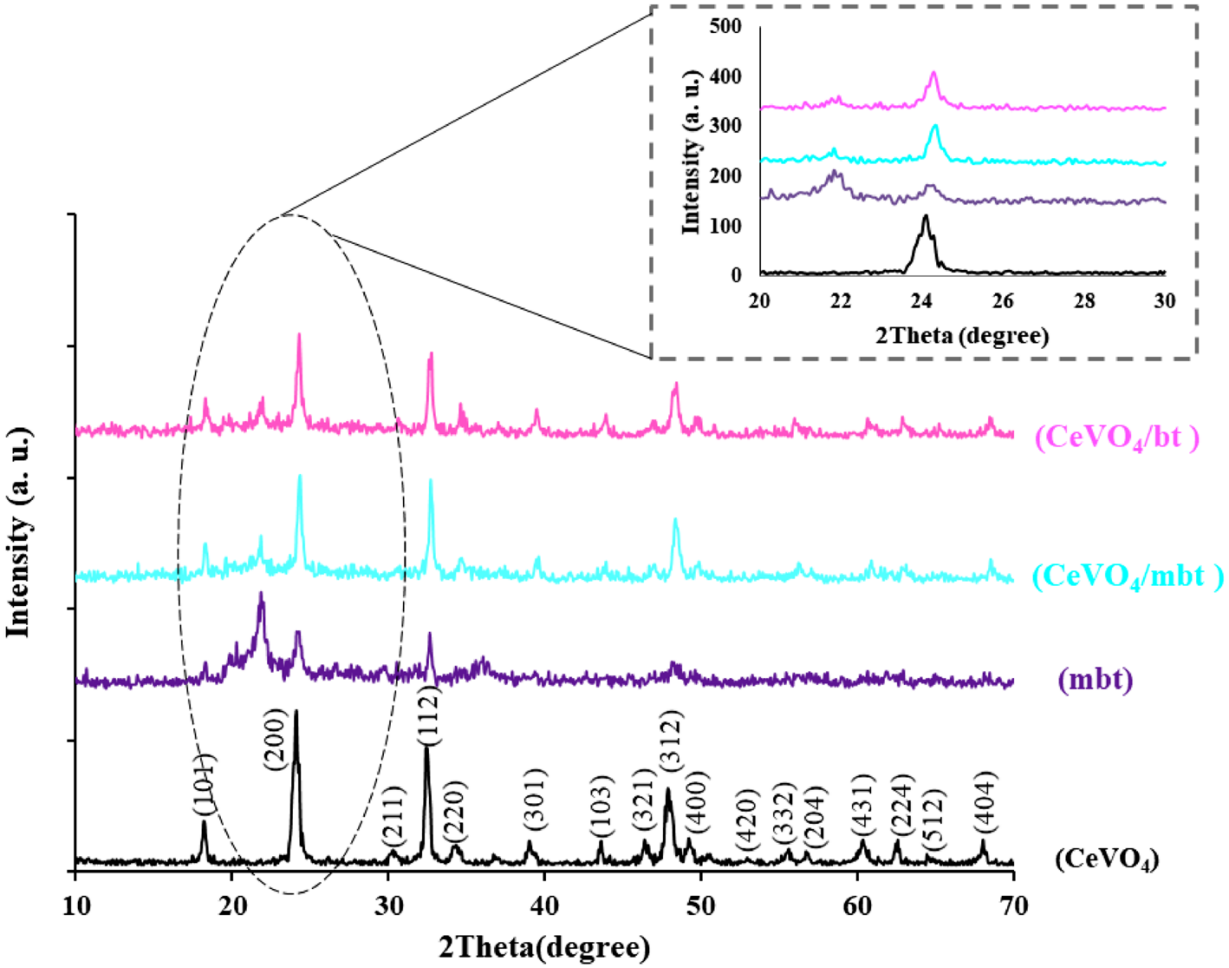
XRD pattern of the as-synthesized products (a) CeVO_4_, (b) modified bentonite (mbt), (c) CeVO_4_/mbt and (d) CeVO_4_/bt.

The purity and crystal structure of CeVO_4_ was investigated using XRD analysis. Based on Fig. 2, the XRD pattern of CeVO_4_ can be attributed to the tetragonal structure with JCPDS = 120,757 and lattice parameters a = b = 7.39 and c = 6.48 Å. The absence of an additional peak in the CeVO_4_ pattern indicates the purity of the CeVO_4_ nanoparticles. Using Scherrer equation^3^, the average crystallite size for CeVO_4_ was calculated to be about 17 nm. In the pattern of the modified bentonite sample, peaks at 21.84, 24.12 and 32.64 degrees have been observed. In the XRD pattern of CeVO_4_/bt and CeVO_4_/mbt composites, the presence of CeVO_4_ and bentonite was validated by its representative diffraction peaks. The appearance of bentonite and CeVO_4_ peaks in the composites is a proof of successful synthesis. With increasing bentonite and modifying CeVO_4_, a slight shift towards higher degrees was observed in XRD pattern of the synthesized composites. This contraction can be due to factors such as the removal of atoms from the crystal lattice or the application of external pressure or strain.

### FT-IR studies

**Figure 3:**
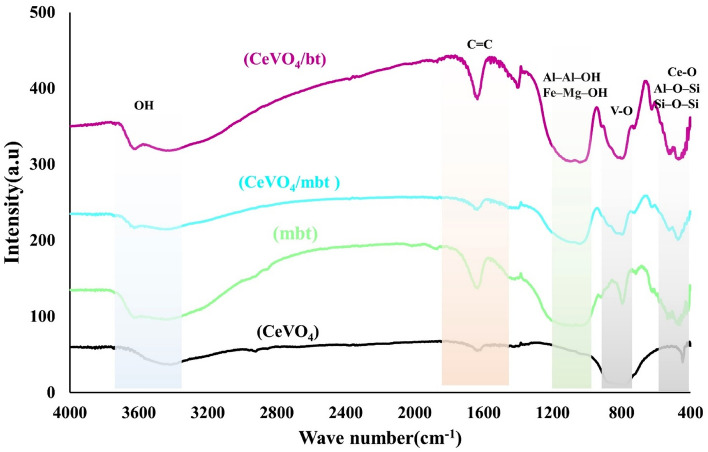
FT-IR spectra of the as-synthesized products (a) CeVO_4_, (b) modified bentonite (mbt), (c) CeVO_4_/mbt and (d) CeVO_4_/bt 0.5

FTIR spectroscopy was applied to investigate the chemical and organic bonds of the as-synthesized samples (Fig. 3). In the FTIR spectrum of CeVO_4_, the peaks at 445 cm^–1^ and 781 cm^–1^, are related to the asymmetric stretching vibrations of Ce–O and V–O^27–30^. In the FTIR spectra of bentonite and modified bentonite, the stretching vibrations of hydroxyl groups (OH) are around 3650 cm^–1^, while the presence of internal OH groups can be seen around 3625, 3445, 1640, 1115, 1030, and 1010 cm^−1^^30,31^. The Si–O–Si and Al–O–Si bending vibrations are associated with the wavenumbers of 500 and 545 cm^−1^^31,32^. Two absorption bands with wavenumber of 915 and 795 cm^−1^ are related to the Al–Al–OH and Fe–OH–Mg bending vibrations, respectively^32,33^.

### FESEM images and EDS-mapping studies

**Figure 4:**
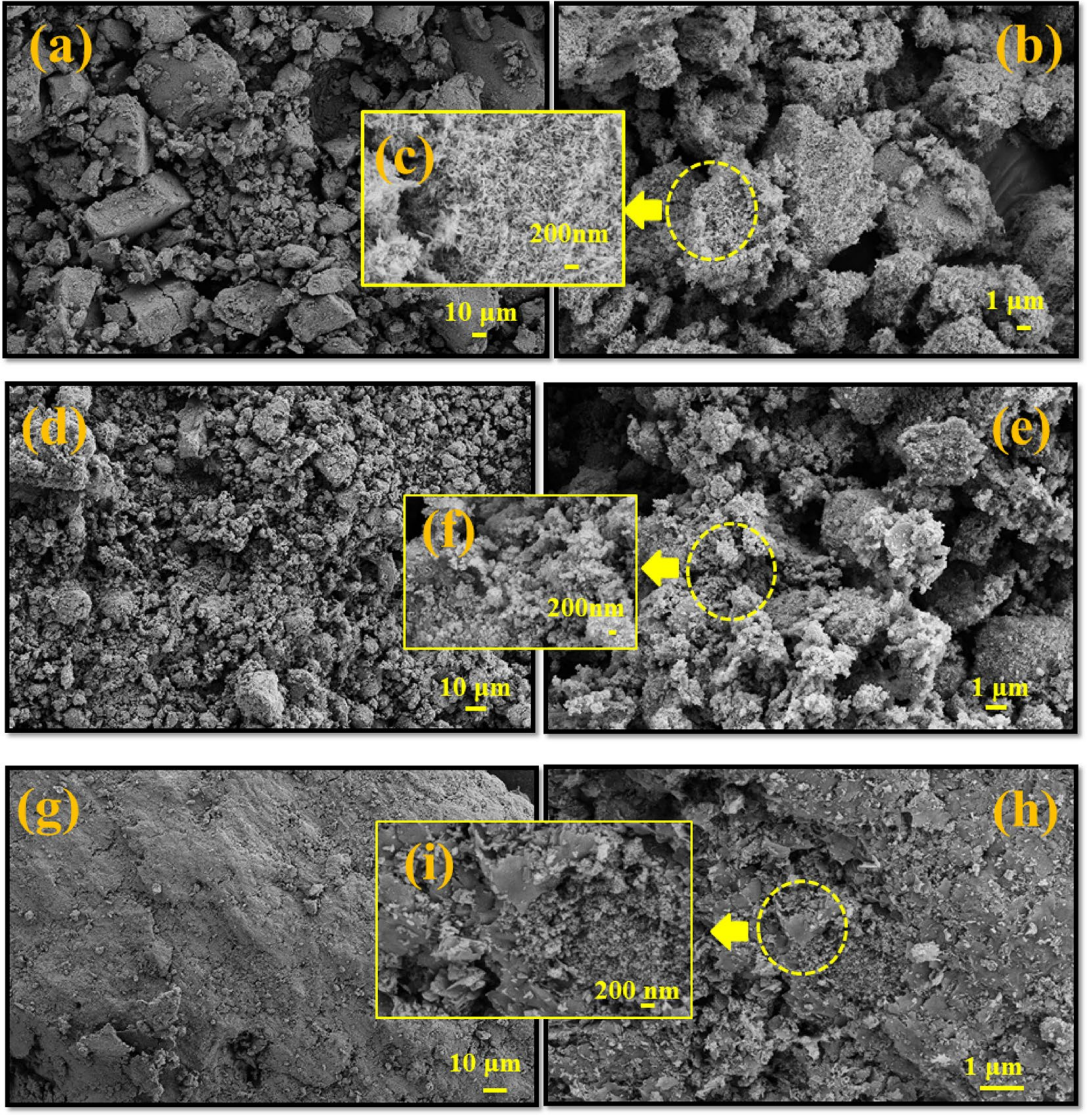
FESEM images of the as-synthesized products (**a-c**) CeVO_4_, (**d-f**) CeVO_4_/bt 0.5 and (**g-i**) CeVO_4_/mbt.

FESEM images were used to examine the morphology of CeVO_4_, CeVO_4_/bt 0.5, and CeVO_4_/mbt samples. In Fig. 4a–c, FESEM images of CeVO_4_ nanoparticles are shown in three different scales. It can be clearly seen that CeVO_4_ nanoparticles have rice-like morphology with a diameter of 20–30 nm and a length of 60–120 nm. To achieve this type of morphology, hydrazine was used because hydrazine controls the particle growth mechanism by controlled release of hydroxide ion^26^. Figure 4d–i show the FESEM images with different magnifications of CeVO_4_/bt 0.5 and CeVO_4_/mbt composites. As seen in the FESEM images of the nanocomposites, CeVO_4_ nanoparticles are well dispersed on the bentonite substrate. It can also be seen that in the presence of bentonite, the size of CeVO_4_ nanoparticles has become smaller, so that the length of the nanoparticles has decreased to about 20–80 nm. The change in the morphology of nanoparticles from rice-like to quasi-spherical is also evident in some places, especially in the case of CeVO_4_/mbt nanocomposite. Furthermore, it can be seen that with the modification of bentonite, the distribution of CeVO_4_ nanoparticles on bentonite has improved.

### EDS studies

**Figure 5:**
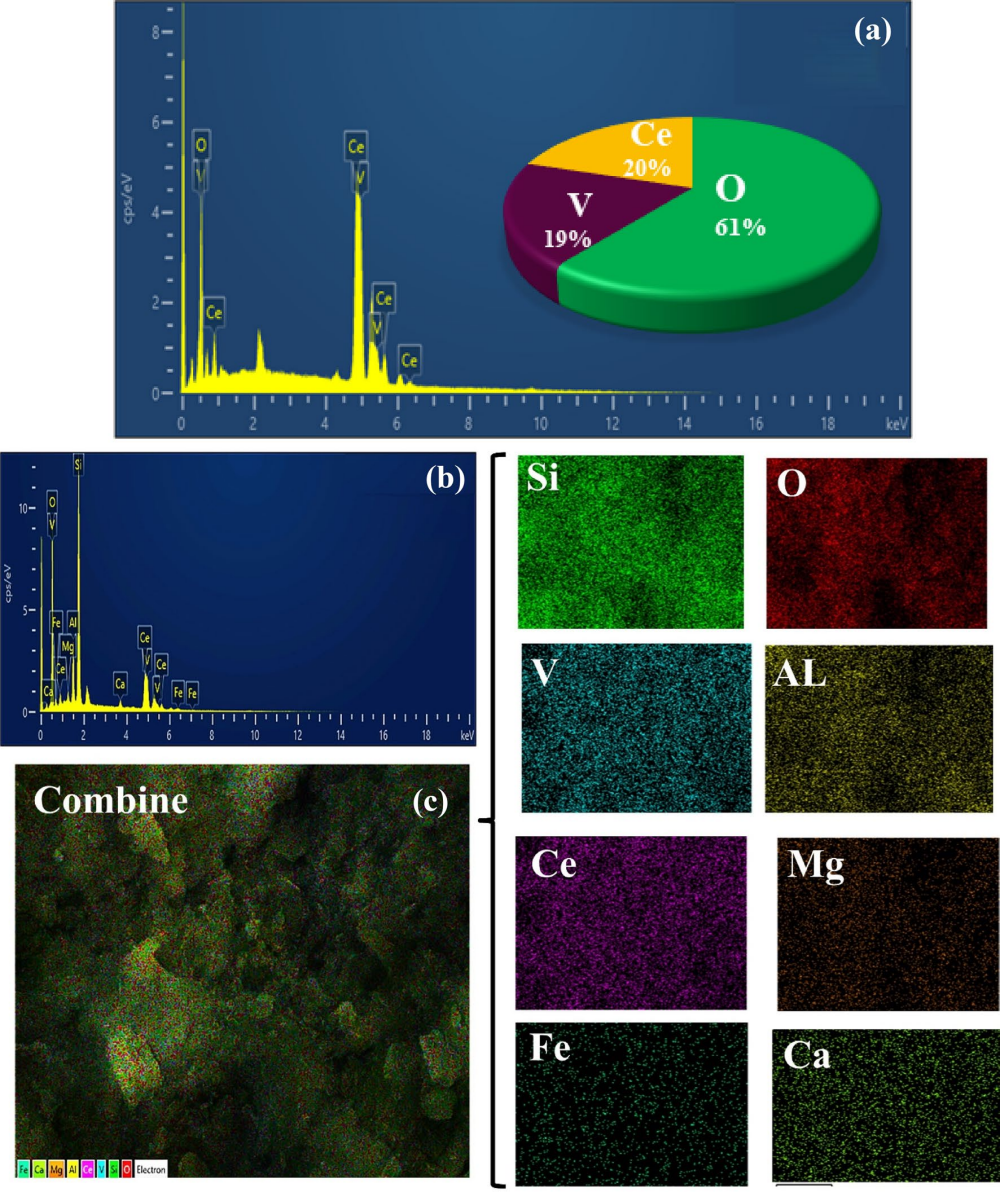
EDS spectra of the as-synthesized products (**a**) CeVO_4_, (**b**) CeVO_4_/bt 0.5 and (**c**) EDS spectrum and elemental analysis mapping of CeVO_4_/mbt nanocomposite.

Quantitative and qualitative analysis of the chemical composition and initial distribution of CeVO_4_ nanoparticles and CeVO_4_/bt 0.5 nanocomposite was investigated using EDS spectroscopy (Fig. 5). According to Fig. 5a, which corresponds to the EDS spectrum of CeVO_4_ nanoparticles, only Ce, V, and O elements are observed and there are no impurities. In the EDS spectrum of CeVO_4_/bt, as shown in Fig. 5b, Ce and V elements can be seen next to the expected elements of bentonite. These results are completely consistent with the XRD results. In order to better investigate the distribution of elements in the compounds and also the distribution of CeVO_4_ nanoparticles on the bentonite substrate, an X-ray map was prepared from the CeVO_4_/modified bentonite composite and presented in Fig. 5c. According to EDS of the as-synthesized nanocomposite, no impurities were observed. Also, the elemental distribution map of CeVO_4_/mbt nanocomposite shows the good dispersion and distribution of CeVO_4_ nanoparticles on bentonite.

### DRS studies

**Figure 6:**
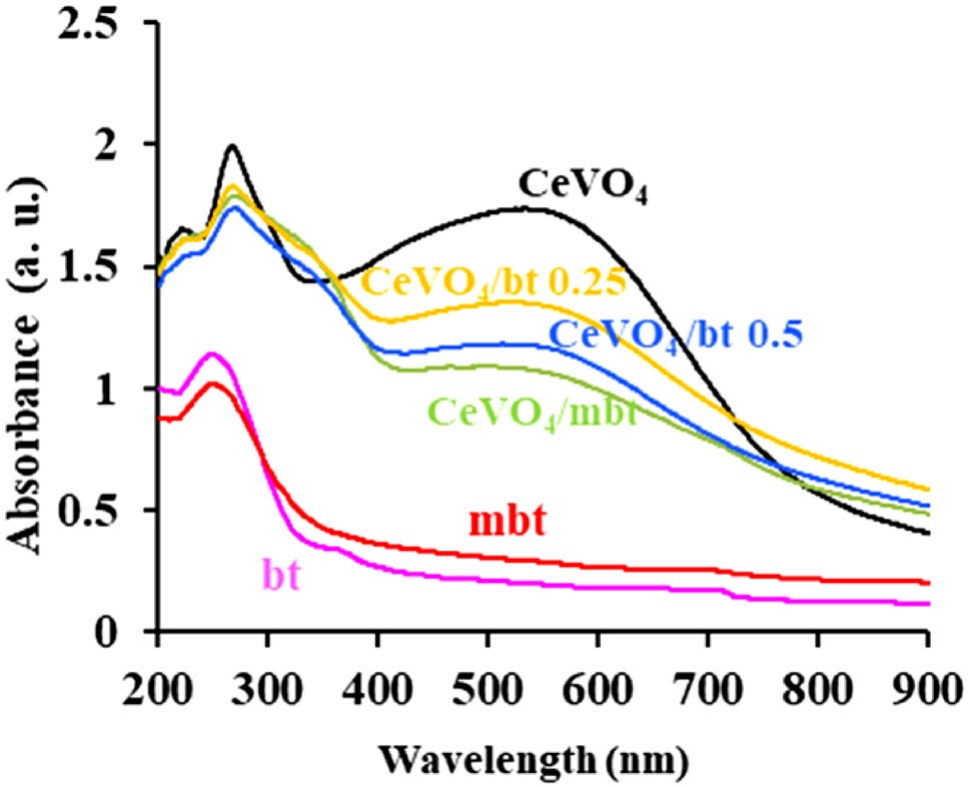
UV–Vis diffuse reflectance spectra of the as-synthesized products.

The light absorption ability of CeVO_4_, bentonite, modified bentonite (mbt), CeVO_4_/bt 0.25, CeVO_4_ /bt 0.5, and CeVO_4_/mbt was studied using reflectance/diffusive transmission (DRS) spectroscopy (Fig. 6). As shown in Fig. 6, cerium vanadate has good absorption in both the visible and ultraviolet regions, and such a result can be expected from cerium vanadate as a photocatalyst material. In contrast, bentonite absorbs little light, especially in the visible region. Along with the surface area, the ability to absorb light is an important parameter in the efficiency of pollutant removal through the photocatalytic process. According to these explanations, it is necessary to investigate the effect of bentonite on the ability of cerium vanadate to absorb light. As can be seen in Fig. 6, cerium vanadate/bentonite nanocomposites (CeVO_4_/mbt and CeVO_4_/bt) have acceptable absorption in visible and ultraviolet light. Therefore, it can be said that the use of bentonite in an optimal amount along with cerium vanadate both increases the surface area for pollutant adsorption and does not significantly decrease the ability to absorb light.

### BET studies

**Figure 7:**
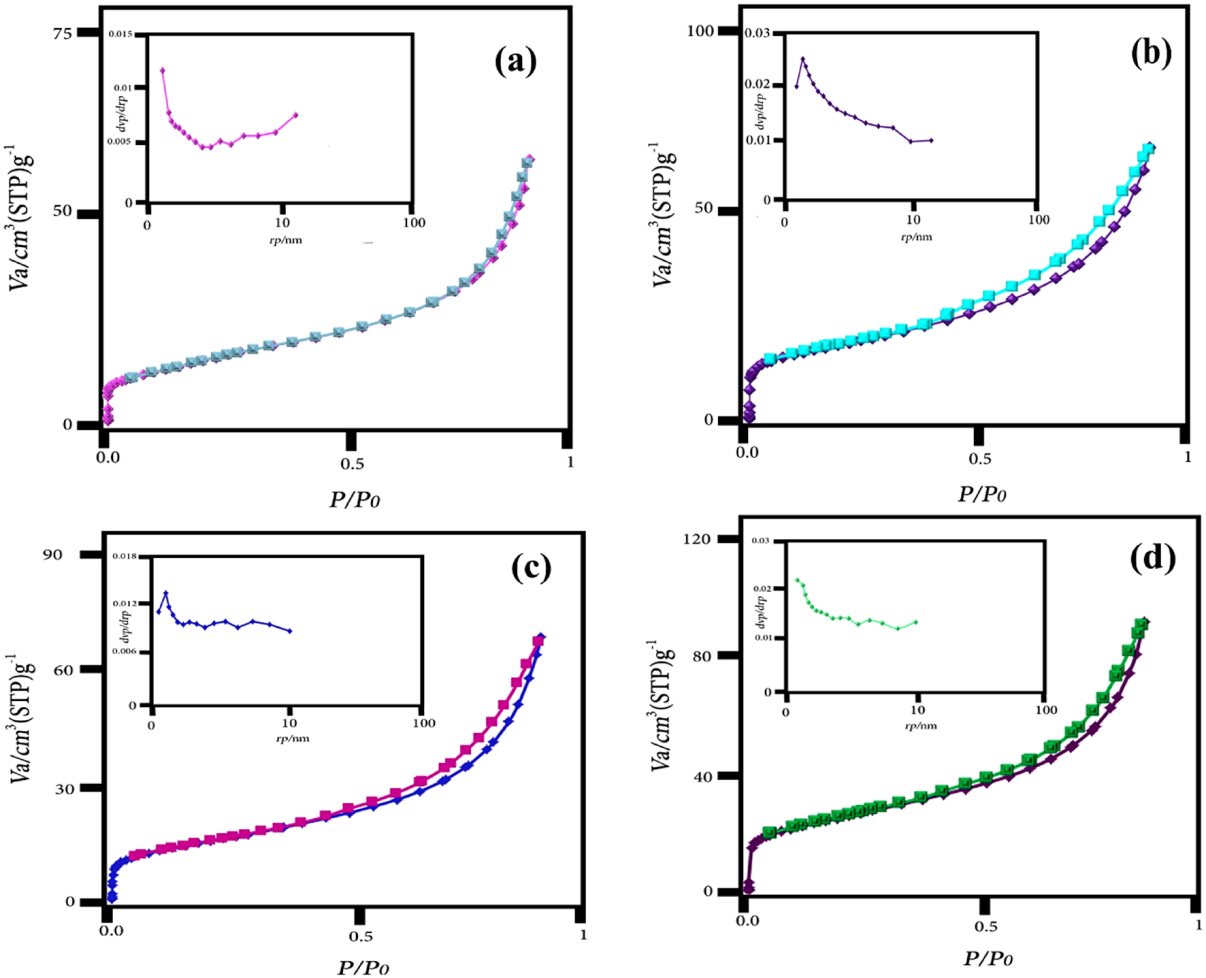
Nitrogen gas adsorption and desorption isotherm and BJH analysis of the as-synthesized products (**a**) CeVO_4_, (**b**) modified bentonite (mbt), (**c**) CeVO_4_/bt, and (**d**) CeVO_4_/mbt.

BET has been used to compare the specific surface area and porosity of the structure of the as-synthesized nanomaterials. Nitrogen adsorption/desorption isotherms and pore size distribution diagrams of CeVO_4_, bentonite, CeVO_4_/bt, and CeVO_4_/mbt are shown in Fig. 7a–d, respectively. The results show that the isotherms of the synthesized products are of type IV with residual loops, which refers to mesoporous materials. Specific surface area, total pore volume, and average pore diameters for the synthesized samples are reported in Table 1. According to Table 1, CeVO_4_/bentonite composite has a higher surface area than pure CeVO_4_. Also, by modifying the bentonite, the surface area is significantly increased. The CeVO_4_/bt composite has a surface area of 50.321 m^2^g^–1^, which has increased significantly after modifying the bentonite surface and reached 91.131 m^2^g^–1^. With these results and considering the surface area as an effective parameter in the pollutant removal process, it can be expected that cerium vanadate/modified bentonite has a high capability as an adsorbent-photocatalyst.

**Table 1:** Specific surface area, average pore diameter, and total pore volume of CeVO_4_, modified bentonite (mbt), CeVO_4_/bt, and CeVO_4_/mbt.

Sample	Specific surface area (m^2^g^–1^)	Total pore volume (cm^3^g^–1^)	Average hole diameter (nm)
CeVO_4_	44.28	0.0861	7.7839
mbentonite	97.816	0.1599	6.5403
CeVO_4_/bt	50.321	0.1008	8.0153
CeVO_4_/mbt	91.131	0.1506	6.6082

### Photocatalytic performance

**Figure 8:**
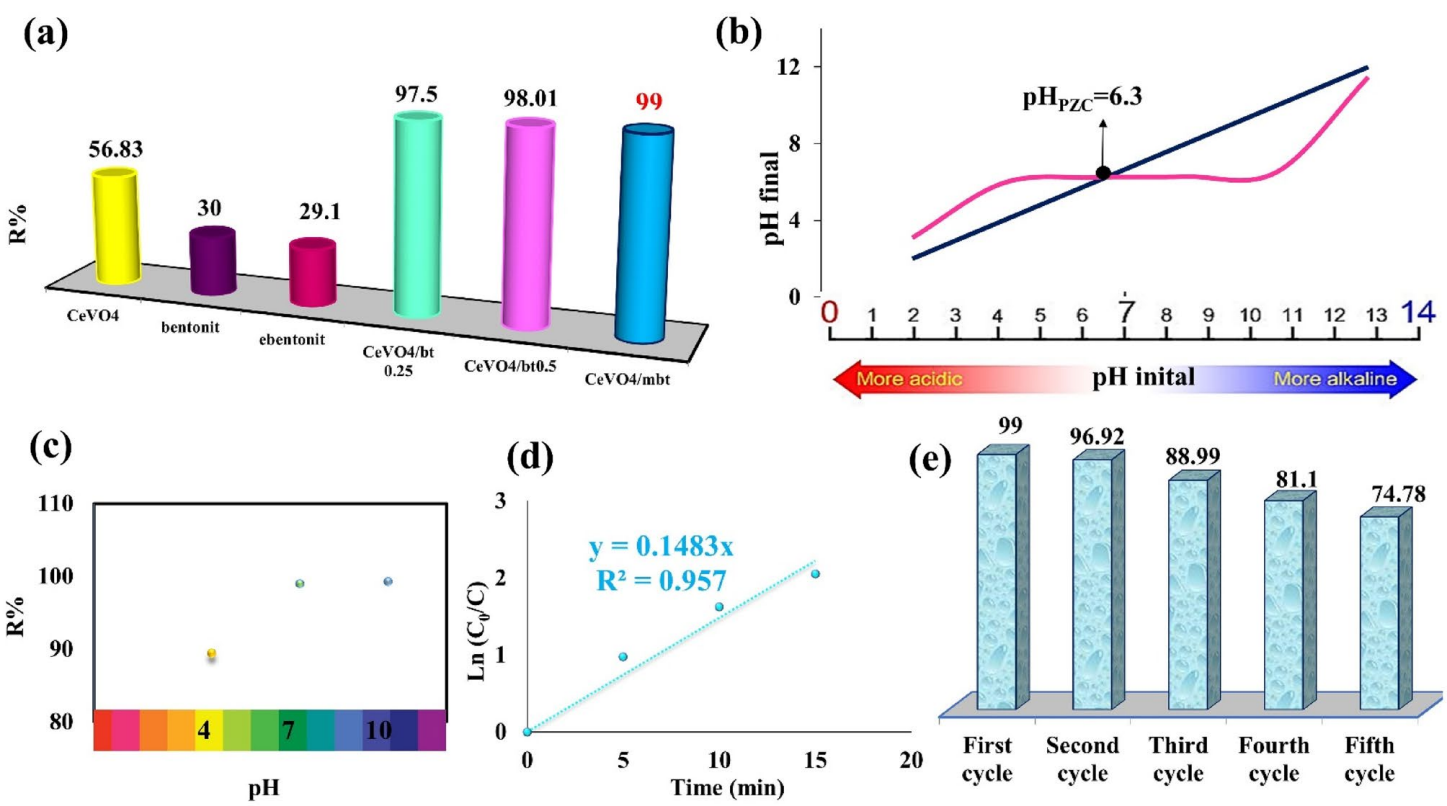
(**a**) Photocatalytic degradation efficiency of methylene blue by CeVO_4_, bentonite (bt), modified bentonite (mbt), CeVO_4_/bt 0.25, CeVO_4_/bt 0.5, and CeVO_4_/mbt, (**b**) point of zero charge (pzc) for CeVO_4_/mbt, (**c**) effect of pH on methylene blue degradation, (**d**) kinetics results of photocatalytic degradation of methylene blue using CeVO_4_/mbt, and (**e**) photocatalytic recyclability of the as-synthesized CeVO_4_/mbt nanocomposite for 5 cycles.

The performance of the as-synthesized products in the photocatalytic degradation of methylene blue using CeVO_4_, bentonite (bt), modified bentonite (mbt), CeVO_4_/bt 0.25, CeVO_4_/bt 0.5, and CeVO_4_/mbt was investigated under visible light irradiation for 15 min (Fig. 8a). As Fig. 8a shows, all nanocomposites (CeVO_4_/bt 0.25, CeVO_4_/bt 0.5, and CeVO_4_/mbt) have better efficiency than pure CeVO_4_ (56.83%), bentonite (30%), and modified bentonite (29.1%). In the comparison between the performance of nanocomposites, CeVO_4_/mbt has shown the best performance (99%). According to BET results, CeVO_4_/mbt has the highest surface area compared to other synthesized nanocomposites. According to the DRS analysis, which determined that the synthesized nanocomposites have almost the same amount of light absorption, it can be said that the higher efficiency of the CeVO_4_/mbt nanocomposite is due to its higher surface area.

### pH effect on methylene blue degradation

The effect of pH on photocatalytic activity can be explicated based on the point of zero charge (pzc), where pH_pzc_ = 6.3 was obtained for CeVO_4_/mbt (Fig. 8b). The photocatalyst surface has a positive and negative charge at pH values lower and higher than pH_pzc_, respectively^34,35^. Therefore, in high pH solutions, the photocatalyst surface has a more negative charge. Thus, the adsorption and finally the destruction of a substance with more positive charge occurs. On the other hand, in solutions with low pH, the surface of the sample is positive and the adsorption of a negatively charged substance occurs more. Three solutions with pHs of 4, 7, and 10 were prepared after adding CeVO_4_/mbt and exposed to visible light for 15 min. It was observed that pH had a significant effect on degradation efficiency. At pH values equal to 7 and 10, which are higher than pH_pzc_, there is an effective interaction between the negatively charged photocatalyst surface and the cationic dye (MB). As a result, as shown in Fig. 8c, higher photocatalytic degradation efficiency was obtained at pH 7 and 10.

### Kinetics study

Figure 8d shows the kinetics results of photocatalytic degradation of methylene blue under visible light irradiation using CeVO_4_/mbt in the test conditions (initial concentration of methylene blue 10 ppm, amount of photocatalyst 0.1 g, pH = 7, and times 0, 5, 10 and 15 min). According to the correlation coefficient of the fitting of the equation, it is observed that the photocatalytic degradation of methylene blue with CeVO_4_/mbt follows the first order kinetics. Also, based on the slope value of the obtained line equation, the methylene blue degradation reaction rate constant was calculated to be 0.1483 min^-1^.

### Reusability experiments of CeVO_4_/mbt

**Figure 9:**
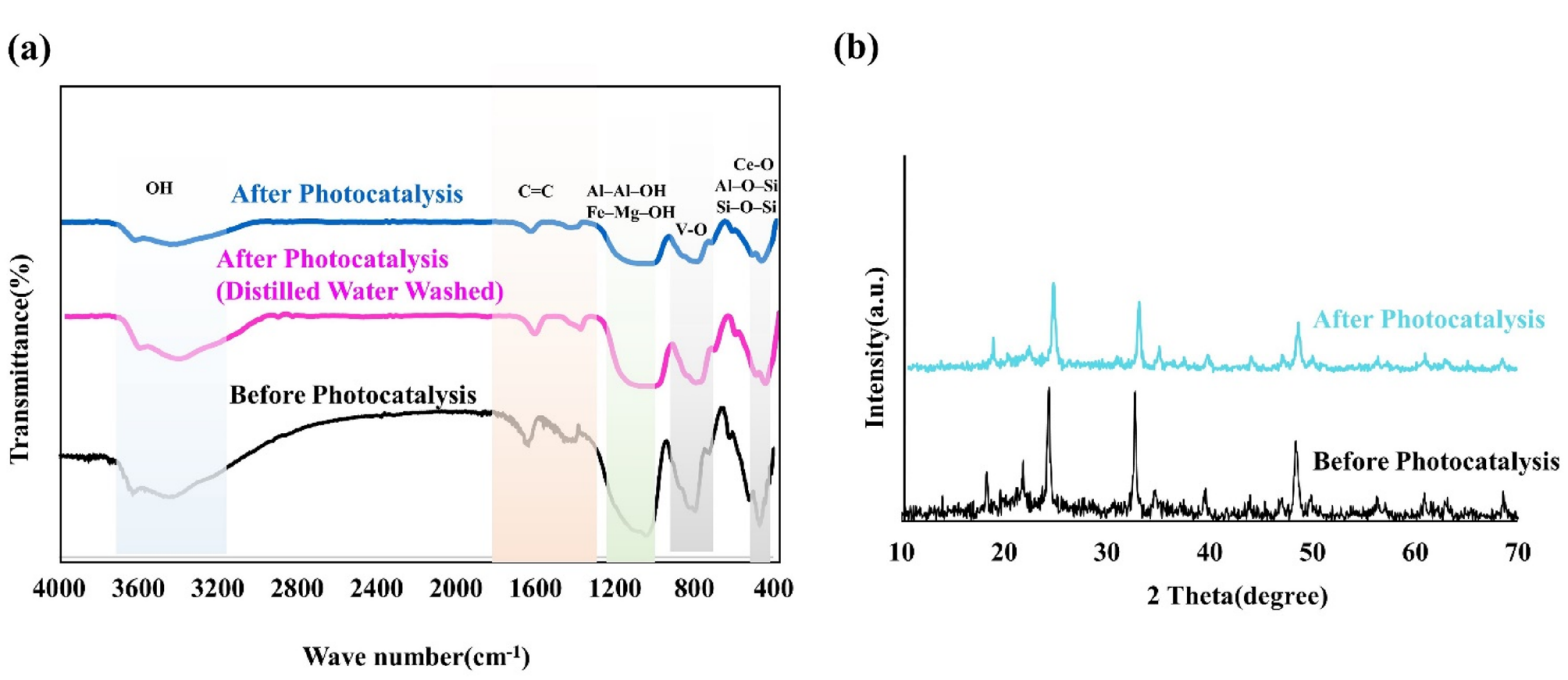
(**a**) FTIR spectra and (**b**) XRD patterns of CeVO_4_/mbt before and after being used for MB photocatalytic degradation.

The reusability of CeVO_4_/mbt photocatalyst in five consecutive cycles is shown in Fig. 8e. The results show that the efficiency drop was not noticeable, which indicates the reusability and acceptable stability of the as-synthesized photocatalyst. Also, Fig. 9 presents the XRD patterns and FTIR spectra of CeVO_4_/mbt before and after being used for MB photocatalytic degradation. It did not show any major change in characteristic and chemical functionality of photocatalyst. The characteristic peaks of CeVO_4_/mbt before and after the photocatalytic degradation of MB are the same with different intensities caused by adsorbed MB, which indicates the stability of sample.

### Mineralization

One common way to measure how well photocatalytic systems work is by total organic carbon (TOC) analysis. Total organic carbon (TOC) analysis measures how much organic carbon is present in a sample. The process of mineralization in water using the produced photocatalysts was assessed by measuring the total organic carbon (TOC), which is the oxygen equivalent of the organic matter in the best sample. The degree of mineralization was calculated using Eq. (2):

2 $$\text{TOC conversion \% }= \frac{{TOC}_{0}-TOC}{{TOC}_{0}}.$$

**Figure 10:**
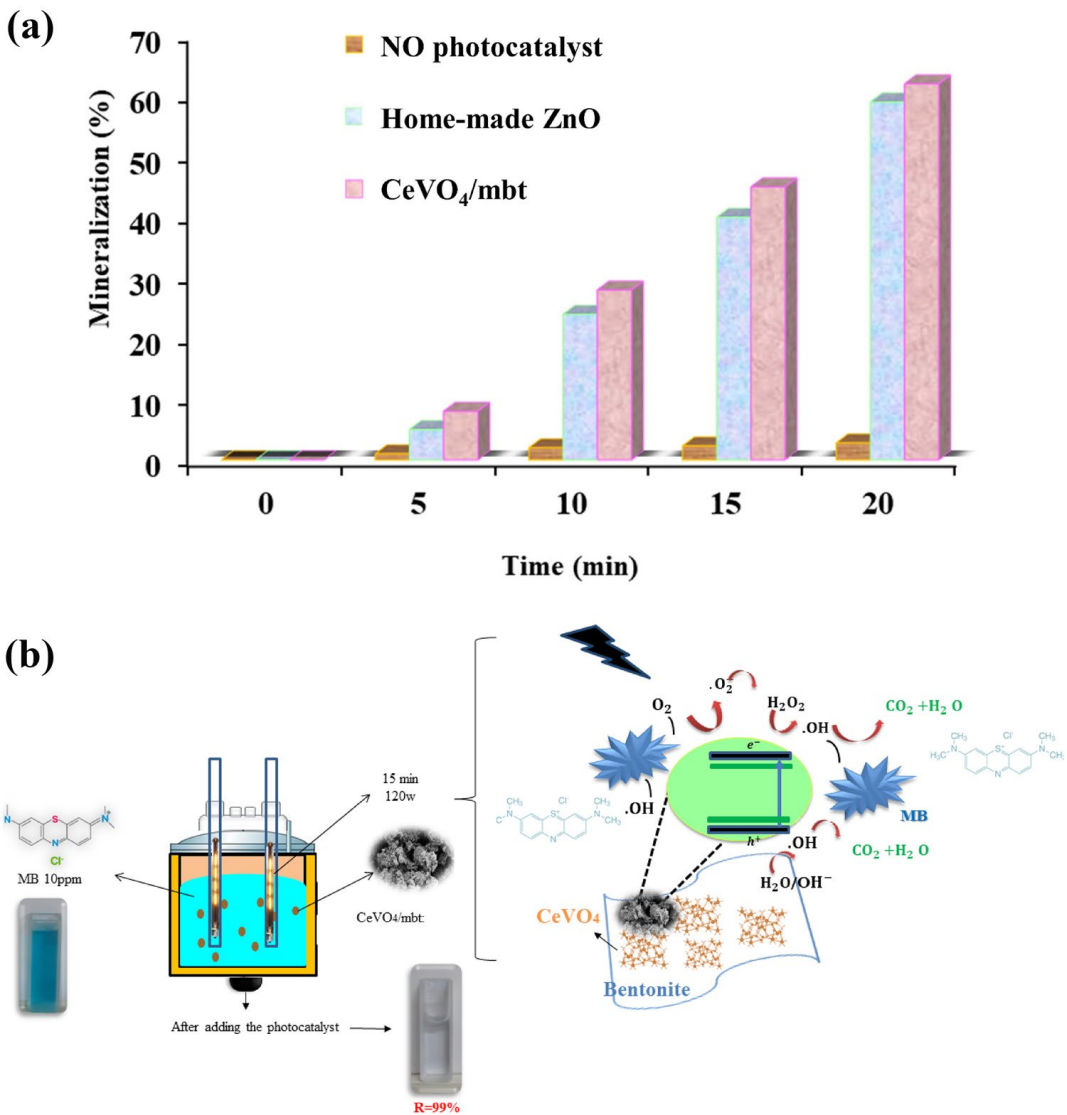
(**a**) Comparison of the mineralization degree (in the presence of CeVO_4_/mbt and no photocatalyst, and home-made ZnO), and (**b**) mechanism of degradation of MB by CeVO_4_/mbt nanocomposite.

In this case, TOC_0_ is the primary TOC of the molecule and TOC is the changed TOC. Figure 10a shows a comparison of the mineralization degree obtained in the presence of no photocatalyst, CeVO_4_/mbt photocatalyst, and homemade ZnO in the test in mixtures (using the same initial pollutant concentration) as a function of irradiation time. The comparison between of data suggests that the mineralization rate of the CeVO_4_/mbt is fully comparable. The MB mineralization were higher in the presence of CeVO_4_/mbt after the same irradiation time, suggesting that this sample had improved photocatalytic activity. It may be inferred from this that the main process of mineralization for MB was the conversion of organic matter into inorganic compounds, such as carbon dioxide and water (Fig. 10b).

### Scavenger test

Light-induced active species play an important role in the photocatalytic process for the rate of degradation, such as hole (h^+^), superoxide anion radical (^**.**^O_2_^–^), and hydroxyl radical (^**.**^OH)^35^. In this regards, the efficiency of photocatalytic degradation of MB after adding sodium chloride, ethanol, and methanol as h^+^, e^–^, and ^**.**^OH scavengers was obtained as 87.5, 87.1 and 86.2%, respectively. Therefore, all three scavengers are almost equally effective in the photocatalytic degradation mechanism of MB, and of course the role of hydroxyl radical is more effective.

As shown in the schematic of Fig. 10b, after the activation of the sample under light irradiation and the excitation of electrons to the conduction band, it is possible to react with oxygen molecules and subsequently superoxide radicals (^**.**^O_2_^–^) will be formed that help to destroy the dye. On the other hand, in the valence band of the photocatalyst, hydroxyl oxidizing radicals are formed from the reaction of h^+^ with water molecules, which play an effective role in destroying dye adsorbed on the surface of the photocatalyst. The following chain reactions have been widely postulated^35–39^.

3 $${\text{CeVO}}_{{4}} /{\text{mbt }} + {\text{ h}}\nu \to {\text{CeVO}}_{{4}}^{*} /{\text{mbt }} + {\text{e}}^{ - } + {\text{h}}^{ + } ,$$

4 $${\text{O}}_{{2}} + {\text{ e}}^{ - } \to^{.} {\text{O}}_{{2}}^{ - } ,$$

5 $$^{ \cdot } {\text{O}}_{{2}}^{ - } + {\text{ H}}^{ + } \leftrightarrow^{.} {\text{OOH,}}$$

6 $${2}^{.} {\text{OOH }} \to {\text{ H}}_{{2}} {\text{O}}_{{2}} + {\text{ O}}_{{2}} ,$$

7 $${\text{H}}_{{2}} {\text{O}}_{{2}} + {\text{ e}}^{ - } \to^{.} {\text{OH }} + {\text{ OH}}^{ - } ,$$

8 $${\text{H}}_{{2}} {\text{O }} + {\text{ h}}^{ + } \to^{.} {\text{OH }} + {\text{ H}}^{ + } ,$$

9 $${\text{OH}}^{ - } + {\text{ h}}^{ + } \to^{.} {\text{OH,}}$$

10 $${\text{R }} + {\text{ h}}^{ + } \to {\text{ R}}^{ + } ,$$

11 $${\text{Dye}}{\mkern 1mu} \, + \,{\mkern 1mu} {\text{radicals}}\,{\mkern 1mu} (^{.} {\text{OH}},^{.} {\text{OOH}}){\mkern 1mu} \, \to {\mkern 1mu} \,{\text{Degradation}}{\mkern 1mu} \rightleftarrows {\mkern 1mu} {\text{product}}{\mkern 1mu} \, \to \,{\mkern 1mu} {\text{CO}}_{2} + {\mkern 1mu} {\text{H}}_{{\text{2}}} {\text{O}}.$$

In order to better express the capabilities of CeVO_4_ based nanocomposites, a brief comparison of recent similar reports and the results obtained in this work is presented in Table 2.

**Table 2:** The brief comparison of recent similar reports and obtained results with this work.

Sample	Brief of obtained results	References
Sb-CeVO_4_	Among all the solutions, 10%-Sb-CeVO_4_ most suitable for the photocatalytic elimination of crystal violet dye (82.6%) in under 35 min	^40^
Pt-CeVO_4_ nanocomposites	The photocatalytic hydrogen generation in Pt/CeVO_4_ nanocomposite was much better compared with pure Pt and CeVO_4_ when exposed to sunlight	^41^
CeVO_4_/rGO nanocomposite	With graphene, the synergistic impact between layers was risen, increasing hydrogen storage capacity. The produced nanocomposite had the maximum hydrogen electrochemical storage of 5430 mAh/g, compared to other nanostructures	^42^
3D pebble-like CeVO_4_/g-C_3_N_4_ nanocomposite	A CeVO_4_/g-C_3_N_4_ nanocomposite was created using a straightforward hydrothermal method; The photocatalyst CeVO_4_/g-C_3_N_4_ significantly improves the breakdown rate of the CIP antibacterial medication to over 90%	^43^
CeVO_4_/Bentonite nanocomposite	Due to the synergistic effects, the MB removal efficiency (for just 0.1 g) of CeVO_4_/mbt nanocomposite was significantly increased (99% in 15 min) compared with neat bentonite, CeVO_4_, and CeVO_4_/bt samples	This study

## Conclusion

In summary, the novel CeVO_4_/bentonite nanocomposite was synthesized through a one-step and controllable hydrothermal method and characterized by XRD, FT-IR, DRS, FESEM, EDS, and BET analyses. To control the growth mechanism and synthesis of composite nanoparticles with rice-like morphology, bentonite was used. The results of the analyses proved the purity of the synthesized CeVO_4_ and showed that rice-like CeVO_4_ nanoparticles were well distributed on the bentonite substrate. Then, the performance of the synthesized nanocomposite in the decolorization of MB dye as a representative of pollutants was evaluated. The results of the photocatalytic process showed that bare cerium vanadate is only able to destroy 50% of methylene blue (under visible light for 15 min), while its composite with bentonite has an efficiency higher than 97%. The reason for this dramatic increase in efficiency is that bentonite dramatically increases the surface area (as an important parameter in the photocatalytic process) while not greatly reducing the amount of light absorption by CeVO_4_. The results showed that by modifying bentonite through ball milling, the pollutant removal efficiency reaches more than 99%, which is an amazing result. Examining the effect of pH showed that the synthesized adsorbent-photocatalyst performs better at a pH higher than 7. Also, the photocatalyst recyclability tests were investigated in 5 cycles, and it was found that the synthesized photocatalyst has good stability.

## Data Availability

The datasets used and analyzed during the current study available from the corresponding author on reasonable request.
